# Quantitative trait locus analysis of parasite density reveals that *HbS* gene carriage protects severe malaria patients against *Plasmodium falciparum* hyperparasitaemia

**DOI:** 10.1186/s12936-015-0920-z

**Published:** 2015-10-07

**Authors:** Maria Rosário do Sambo, Carlos Penha-Gonçalves, Maria Jesus Trovoada, João Costa, Roberto Lardoeyt, António Coutinho

**Affiliations:** Instituto Gulbenkian de Ciência, Oeiras, Portugal; Hospital Pediátrico David Bernardino, Luanda, Angola; Faculdade de Medicina, Universidade Agostinho Neto, Luanda, Angola; Centro Nacional de Endemias, São Tomé, São Tomé and Príncipe; Faculdade de Medicina, Universidade Katyavala Bwila, Benguela, Angola

**Keywords:** Malaria, Sickle cell, Hyperparasitaemia, Severe malaria, *HbS*, Angola

## Abstract

**Background:**

Haemoglobin S (*HbS*) is the gene known to confer the strongest advantage against malaria morbidity and mortality. Multiple *HbS* effects have been described resulting in protection against parasitaemia and reduction of severe malaria risk. This study aimed to explore *HbS* protection against severe malaria and *Plasmodium falciparum* parasitaemia in Angolan children exhibiting different severe malaria syndromes.

**Methods:**

A case–control study was designed with 430 malaria cases (*n* = 288 severe malaria and *n* = 142 uncomplicated malaria) and 319 uninfected controls, attending a central paediatric hospital in Luanda. Severe malaria syndromes were cerebral malaria (*n* = 130), severe malaria anaemia (*n* = 30) and hyperparasitaemia (*n* = 128). Quantitative trait locus analysis was carried out to study *HbS* association to parasite densities.

**Results:**

Previously reported *HbS* protection against severe malaria was confirmed in case–control analysis (*P* = 2 × 10^−13^) and corroborated by transmission disequilibrium test (*P* = 4 × 10^−3^). High parasite density protection conferred by *HbS* was detectable within severe malaria patients (*P* = 0.04). Stratifying severe malaria patients according parasite densities, it was found that *HbS* was highly associated to hyperparasitaemia protection (*P* = 1.9 × 10^−9^) but did not protect non-hyperparasitaemic children against severe malaria complications, namely cerebral malaria and severe malaria anaemia. Many studies have shown that *HbS* protects from severe malaria and controls parasite densities but the analysis further suggests that HbS protection against severe malaria syndromes was at a large extent correlated with control of parasitaemia levels.

**Conclusions:**

This study supports the hypothesis that *HbS* confers resistance to hyperparasitaemia in patients exhibiting severe malaria syndromes and highlights that parasitaemia should be taken into account when evaluating *HbS* protection in severe malaria.

## Background

The haemoglobin S mutation (*HbS*) is very well recognized as the human single gene conferring the strongest advantage against falciparum malaria [1, 2]. Evidence from *HbS* genetic association studies in African countries with malaria endemicity levels, helped to settle the notion that epidemiology of sickle-cell trait results from the selective pressure imposed on the human genome by *Plasmodium* infection [3–8]. Multiple biochemical and immune mechanisms have been suggested to explain *HbS* protection against *Plasmodium falciparum* pathogenesis and morbidity. It is proposed that such mechanisms act concurrently to improve parasitaemia control and protect from uncomplicated and severe malaria [9].

In Angola, sickle-cell disease is a critical public health concern and is considered a main contributor to the high mortality rate in children [10, 11]. However, there are few reports on *HbS* prevalence in Angolan populations [12–14]. Malaria is one of the leading causes of morbidity and mortality in Angola, mainly among preschool children, and it is estimated that *P. falciparum* causes 90 % of all malaria infections [11, 15–17].

This study aimed to analyse the role of *HbS* mutation in malaria protection in Angolan children, according to disease severity and degree of infection. The results of this stratified analysis suggest that *HbS* carriers exhibiting severe malaria syndromes are significantly protected against hyperparasitaemia.

## Methods

### Place of study and ethical permission

Luanda is an endemic-malaria province with high level of transmission [18]. Hospital Pediátrico David Bernardino (HPDB) is a tertiary national reference paediatric hospital in Luanda, Angola. Ethical permission for this study was granted by the Ethics Committee of the HPDB in Luanda that was appointed by the Angolan Ministry of Health.

### Subjects

A total of 749 children, living in Luanda and ranging from 6 months to 13 years of age, were enrolled in the present study. Cases and controls were selected among attendance to the HPDB. The sample collection was carried out from February 2005 to May 2007 and comprised 288 severe malaria children (130 with cerebral malaria and 158 with severe malaria but not cerebral malaria), 142 patients with uncomplicated malaria and 319 uninfected controls. Samples were also collected from mothers of severe malaria children and comprised 226 mother–child pairs. Mothers were enrolled only after written, informed consent and children were enrolled only after written, informed consent from their parents or guardians.

### Phenotypic and inclusion criteria

Malaria was diagnosed on the basis of a positive asexual parasitaemia detected by a single reader on a Giemsa-stained thick film. For parasitaemia quantification 100 high-power microscopic fields were observed. The number of asexual parasites and white blood cells (WBCs) were counted in each field until the number of WBCs reached 200 and the parasite density was calculated from this value [19]. To confirm and select for *P. falciparum* infection, children with mixed infections, as ascertained by malaria species-specific nested-PCR in peripheral blood DNA [20], were excluded from the study. Triage and clinical examinations of patients with severe disease included a clinical history collected upon admission to the paediatrics urgency ward, followed by detailed clinical examination as described below.

### Clinical criteria

The diagnosis of cerebral malaria followed the criteria of the WHO definition, strictly for research purposes, valuing unarousable coma, which was defined by the inability to localize painful stimuli in absence of other causes of encephalopathy [21]. For the purposes of this study, whenever coma has been preceded by a seizure the assessment of coma was made 1 h after the end of the seizure. This procedure is meant to exclude transitional post-convulsive coma.

The cerebral malaria patients had to meet all the following criteria: (1) coma score <3 in the Blantyre Scale, for children younger than 60 months or score <7 on the Glasgow Scale, for children aged equal or more than 60 months; (2) absence of diagnostic criteria for any other possible cause of encephalopathy, including hypoglycaemia (venous blood glucose below 40 mg/dL); (3) exclusion of meningitis-cerebrospinal fluid without pleocytosis (up to 8 lymphocytes/cu mm) nor hypoglycorrachia (determination of glucose in cerebrospinal fluid >50 mg/dL or equal to 50 % of blood glucose, never <40 mg/dL).

Severe malaria anaemia (SMA) was defined as haemoglobin <5 g/dL or haematocrit <15 % and hyperparasitaemia (HP) required a value of parasitaemia >100 parasitized erythrocytes per microscopic field (magnification 1000×). The presence of any diagnosis or any other neurological sign, including seizures, installed during the course of the disease excluded the child from the study.

Uncomplicated malaria patients had no clinical manifestations suggestive of complications of malaria, as well as any other pathology that could explain a febrile syndrome.

Controls were uninfected children randomly selected in the vaccination ward of the HPDB. Children in this group had no symptom of disease and were negative in a PCR test for *Plasmodium* DNA performed in peripheral blood.

### Blood DNA preparation

DNA was extracted from peripheral blood using the Chemagen Magnetic Bead Technology in an automated nucleic acid isolation station. DNA preparations were quantified using PicoGreen reagents (Invitrogen^®^) according to the supplier instructions. The dbSNP, rs334, that defines the *HbS* mutation, was genotyped with the Mass Array system to design multiplex reactions for PCR and iPlex primer extension (Sequenom) and the MALDI-TOF based Mass Array genotype platform (Sequenom). Genotyping rate was higher than 90 %.

### Phenotypic data analysis

The statistical analysis for sample characterization was performed with SPSS version 15.0. The data that were not normally distributed were analysed using non-parametric methods (Mann–Whitney U and Kruskal–Wallis) and Pearson Chi square was used to study the association between qualitative variables. A threshold for statistical significance was *P* < 0.05; odds ratios (OR) and 95 % confidence interval were calculated to measure the magnitude of association.

Logistic regression was performed with parasitaemia as a dependent variable dichotomized as <100 and ≥100 parasitized erythrocyte/microscopic field using diagnosis and age group in bivariate analysis.

### Genetic analysis

Hardy–Weinberg equilibrium requirements (*P* > 0.05) were met in uninfected controls. Case–control association analysis was performed with the logistic regression model implemented in the SNPassoc package for R software (version 2.7.0) using allelic and genotypic frequencies where dichotomized disease outcomes were analysed as variables dependent on presence of allele T or presence of genotype AT. Genotypic analysis used the dominant and additive genetic models which are different from the actual underlying mode of inheritance of the minor allele [22]. The significance level of the likelihood ratio test *P* < 0.05 was considered as suggestive evidence for association. TRANSMIT software that allows TDT testing when phase is unknown was used [23] to analyse *HbS* transmission in mother–child pairs. Quantitative trait analysis (QTL) of parasite densities was performed with the program Plink (version 1.06) that calculates the level of significance, either by using the asymptotic model, or the empirical model [24]. QTL analysis considered the additive linear model where the parasite density is analysed as a continuous variable dependent on the *HbS* genotype status (AA, AT and TT).

## Results

### Sample characterization

**Fig. 1:**
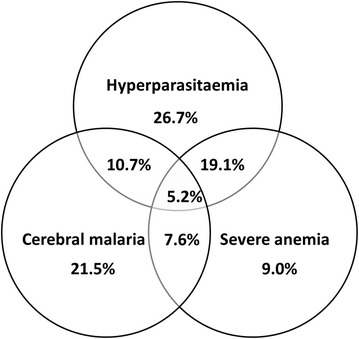
Co-occurrence of malaria complications in severe malaria patients. Venn diagram representing the overlap of specific clinical malaria manifestations (cerebral malaria, hyperparasitaemia and severe anaemia) in 288 severe malaria patients

In this hospital-based study, association analyses of the *HbS* mutation (dbSNP rs334) was performed in Angolan children with distinct malaria phenotypes with the primary purpose of stratifying the *HbS* genetic effect according malaria clinical traits. The study entailed 430 malaria patients and 319 uninfected control children. All patients were enrolled at the HPDB in Luanda and were clinically evaluated as described in the “Methods”. The patient group was composed of 142 patients with uncomplicated malaria (UM) phenotype and 288 patients with severe malaria (SEV). When analyzing the clinical phenotype of the SEV patients it was found that only a part of them had criteria for defining cerebral malaria (CM), which justified the separation into two distinct groups, such as CM (*n* = 130) and severe malaria with SMA and/or HP but no symptoms of CM (*n* = 158). However, in each group it was found that there was overlap of additional complications identified as follows: CM/SMA (7.6 %), CM/HP (10.7 %), SMA/HP (19.1 %), and CM/SMA/HP (5.2 %) (Fig. 1). Within the group of SEV patients 202 had hyperparasitaemia and 86 had low parasitaemia levels (nHP). Patients with UM were outpatients, whereas the SEV patients were hospitalized. Uninfected controls (UIF) were recruited from the Department of Vaccination of HPDB that proceeded from the same population.

**Fig. 2:**
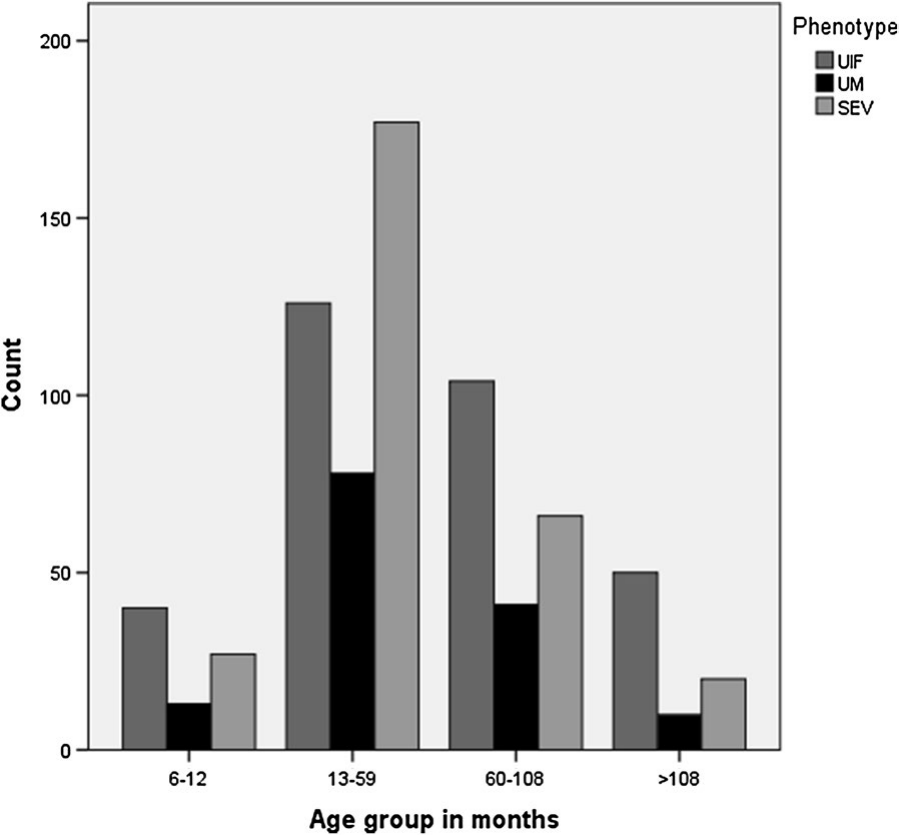
Clinical phenotypes and age groups in months

Kimbundu, the predominant ethnic group in Luanda was the most represented in the sample (50.7 %) followed by the Umbundu group (13.7 %). This ethnic representation did not differ in UM, SEV and UIF patient groups (Chi square test, *P* = 0.46). Although male gender prevailed in all groups (Table 1), no significant sex bias was found across malaria and control groups (Chi square test, *P* = 0.17). The distribution of age groups in SEV and UM patients (Fig. 2) were comparable with median values of 43 and 39.5 months, respectively. No significant differences were found in age distribution of SEV, UM and UIF groups as evaluated by the Mann–Whitney U-test (*P* = 0.14). Median value of parasite densities was higher in the age group 13–59 months and multiple comparison by Kruskall–Wallis test revealed that parasitaemia densities differed across age groups (*P* = 0.003). However logistic regression with parasitaemia and age groups was not significant (*P* = 0.10). The fatality rate in SEV patients was 13 % and was significantly higher in CM patients as compared to the severe non-cerebral malaria cases (Chi square test, *P* = 3 × 10^−3^), [OR 1.89 (2.33–1.54)].

**Table 1:** Gender representation in malaria patients and uninfected controls

Gender	Severe malaria	Uncomplicated malaria	Uninfected controls	Total
Male (n) (%)	172 (59.7 %)	75 (52.8 %)	179 (56.1 %)	426 (56.9 %)
Female (n) (%)	116 (40.3 %)	67 (47.2 %)	140 (43.9 %)	323 (43.1 %)
Total	288	142	319	749

### *HbS* effects in clinical malaria

The role of the *HbS* mutation in different clinical malaria traits was studied to determine whether the sample replicated the expected malaria protective effect. Predictably, the T allele at dbSNP rs334 (*HbS* mutation) was significantly under-represented in SEV patients (3.7 %) as compared to UM patients (10.9 %) and to UIF controls (15.1 %) (Table 2). These allelic differences were reflected on the heterozygous genotype frequency that was remarkably low in severe malaria (1.5 %) as compared to age-matched general population (23.7 %) suggesting that sickle cell trait had a stronger protection effect against SEV [OR 0.15 (0.09–0.27)] than against UM [OR 0.50 (0.30–0.84)] (Table 2). The transmission disequilibrium test (TDT) showed a significant reduction in transmission of *HbS* allele (T allele) to children with SEV (*P* = 0.002) (Table 3). The TDT result supported the notion that absence of *HbS* favours the development of SEV and corroborates the results of case–control analysis lessening the possibility of population stratification effects. It is noteworthy that *HbS* conferred strong protection against CM and against non-cerebral forms of SEV, suggesting that protection against SEV was irrespective of CM development (Table 2).

**Table 2:** *HbS* allelic and genotypic frequency (AT for dbSNP rs334) and genetic association to uncomplicated and severe malaria

Genetic testing	SEV	UM	UIF	UM/UIF	SEV/UIF	CM/UIF	SnC/UIF
	n = 263 (%)	n = 141 (%)	n = 304 (%)	*P*	*P*	*P*	*P*
				OR (95 % CI)	OR (95 % CI)	OR (95 % CI)	OR (95 % CI)
Allelic	3.7	10.9	15.1	9 × 10^−2^	6 × 10^−11^	9 × 10^−4^	3 × 10^−6^
				0.69 (0.45–1.07)	0.22 (0.13–0.36)	0.26 (0.11–0.61)	0.31 (0.17–0.54)
Genotypic^a^	1.5	8.5	23.7	7 × 10^−3^	2 × 10^−13^	1 × 10^−9^	4 × 10^−8^
				0.50 (0.30-0.84)	0.15 (0.09-0.27)	0.11 (0.04-0.28)	0.19 (0.30-0.84)

The numbers for SEV, UM and UIF represent only those with successful genotyping

*SEV* severe malaria, *UM* uncomplicated malaria, *UIF* uninfected, *CM* cerebral malaria, *SnC* severe non-cerebral malaria

^a^Performed with the dominant genetic model comparing A/A genotypic frequencies to A/T and T/T frequencies

**Table 3:** *HbS* transmission disequilibrium test in 226 mother-severe malaria child pairs

Allele	Observ	Expec	Var (O–E)	P value
T	20	30.4	9.9	0.001
A	432	421.6	9.9	0.001

*Observ* observed transmissions, *Expec* expected transmissions, *Var (O–E)* variance (observed-expected)

### *HbS* controls parasite density in severe malaria

**Fig. 3:**
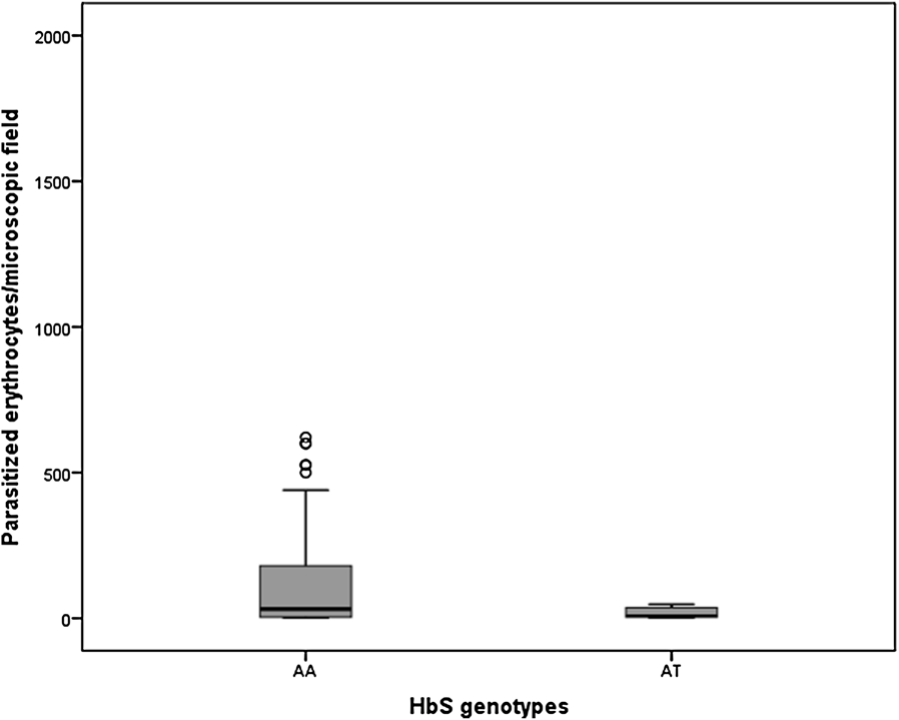
Parasitaemia distribution stratified by *HbS* genotype. *Plot* represents parasite densities in all malaria patients (n = 288). *HbS* genotypes are represented heterozygous (AT) and non-carriers (AA). Parasite density represents the number of parasitized erythrocytes/microscopic field. Data are represented in *boxes* that include 50 % of sample distributions (2nd and 3rd quartiles) with *horizontal lines* (median) and outliers are represented by *circles*

**Fig. 4:**
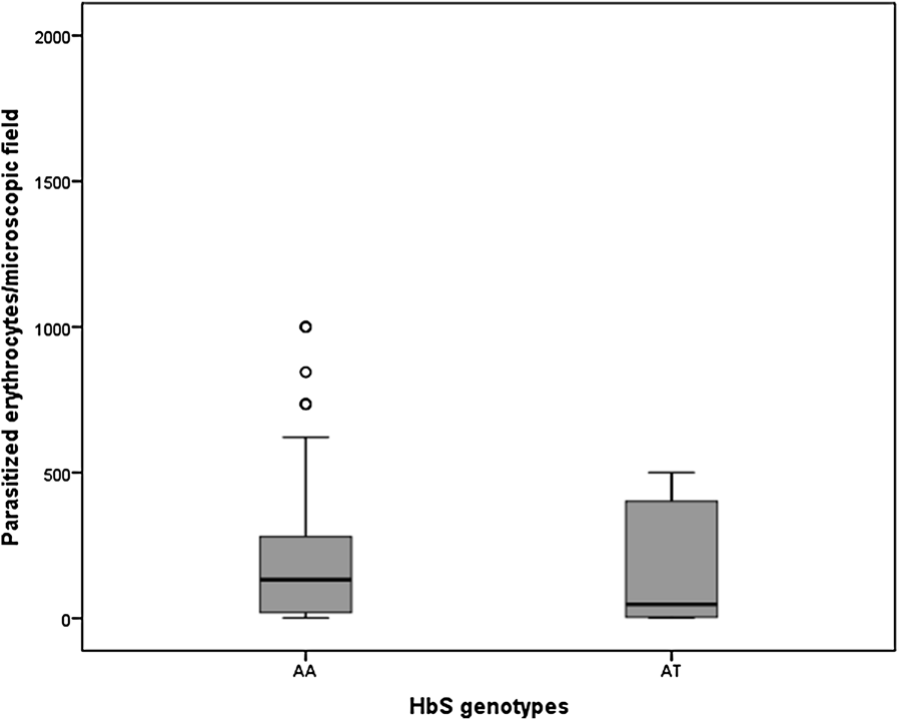
Parasitaemia distribution stratified by *HbS* genotype. *Plot* represents parasite densities only in the sub-set of severe malaria patients (cerebral malaria, severe anaemia and hyperparasitaemia). *HbS* genotypes are represented heterozygous (AT) and non-carriers (AA). Parasite density represents the number of parasitized erythrocytes/microscopic field. Data are represented in *boxes* that include 50 % of sample distributions (2nd and 3rd quartiles) with *horizontal lines* (median) and outliers are represented by *circles*

Next, it was explored whether *HbS* protection against SEV correlated with control of parasitaemia levels. Median parasitaemia levels in all malaria patients (UM + SEV) were lower in presence of *HbS* (Fig. 3) and after UM exclusion this effect was also observed in SEV patients (Fig. 4). This observation was corroborated by QTL analysis as it was found that *HbS* best fitted an additive mode of action in the reduction of the parasite density in children with malaria, irrespective of disease severity (Table 4). Albeit the low *HbS* frequency in SEV children reduces statistical power, the QTL analysis has detected a significant *HbS* effect in protecting SEV patients against hyperparasitaemia (Table 4).

**Table 4:** Parasite density QTL analysis for *HbS*

Patients	BETA	L95	U95	P value
UM + SEV	−87.57	−142.3	−32.84	0.002
SEV	−127.1	−209.1	−45.09	0.04

The results refer to QTL analysis using the additive model

BETA is the regression coefficient; confidence intervals (L95 and U95)

*UM* uncomplicated malaria, *SEV* severe malaria

To determine whether the protection against SEV afforded by *HbS* was conditioned to the parasitaemia status, SEV patients were grouped as hyper-parasitaemic (HP, parasitaemia ≥100) and non-hyperparasitaemic (nHP, parasitaemia <100), irrespective of other observed clinical complications.

*HbS* allelic frequency was higher in non-hyperparasitaemic children suggesting that *HbS* protects SEV patients from developing hyperparasitaemia (Table 5). Thus, seven out of nine SEV patients carrying the *HbS* allele did not show hyperparasitaemia.

**Table 5:** *HbS* allelic and genotypic frequency (TA for dbSNP rs334) and severe malaria association *HbS* conditioned to parasitaemia levels

Genetic testing	HP	nHP	HP/UM	nHP/UM	HP/UIF	nHP/UIF
	n = 178 (%)	n = 110 (%)	*P*	*P*	*P*	*P*
			OR (95 % CI)	OR (95 % CI)	OR (95 % CI)	OR (95 % CI)
Allelic (T)	1.1	6.4	1.8 × 10^−7^	0.13	4.9 × 10^−9^	0.16
			0.11 (0.04–0.30)	0.53 (0.24–1.21)	0.10 (0.04–0.26)	0.67 (0.38–1.18)
Genotypic^a^	1.1	1.8	1 × 10^−4^	0.23	1. 9 × 10^−6^	1. 6 × 10^−2^
			0.19 (0.08–0.48)	0.55 (0.21–1.45)	0.11 (0.04–0.27)	0.42 (0.20–0.85)

The numbers for HP and nHP represent only those with successful genotyping

*HP* hyperparasitaemia, *nHP* non-hyperparasitaemia, *UM* uncomplicated malaria, *UIF* uninfected

^a^Performed with the dominant genetic model comparing A/A genotypic frequencies to T/A and T/T frequencies

Allelic association analysis stratified for parasitaemia showed that *HbS* strongly protects against SEV occurring with HP but did not confer overt advantage against SEV in nHP children (Table 5). Together, these results imply that strong protection afforded by *HbS* against severe malaria in this cohort is coupled to hyperparasitaemia resistance and further suggest that other factors are controlling susceptibility/resistance to non-hyperparasitaemic severe malaria.

## Discussion

The mechanism of *HbS* protection against *P. falciparum* morbidity remains controversial [25–27]. Studying a sample of Angolan children with different malaria syndromes collected evidence showed that *HbS* conferred specific protection against hyperparasitaemia, specifically in the context of severe malaria. In this study population, a significant number of malaria patients (42 %) exhibited concomitantly different malaria clinical complications. These overlapping malaria syndromes corroborate the notion that severe malaria cases often represent clinical syndromes rather than clinical isolated entities [28]. In this context, studying *HbS* protection against one clinical condition faces possible confounding by co-occurrence of other infection parameters. Strikingly, by contrasting allelic and genotypic *HbS* frequencies in severe malaria patients stratified either for parasitaemia values or by other causes of clinical severe malaria (namely, cerebral malaria and/or severe malaria anaemia), it was found that protection against severe malaria was at a large extent attributable to resistance to hyperparasitaemia. Although with limitations due to the reduced number of *HbS* homozygotes the analysis supports the notion that control of parasite density was quantitatively dependent on the number of T alleles.

Furthermore, *HbS* showed to control quantitatively parasitaemia in clinical malaria both in presence or absence of uncomplicated malaria cases. This result suggests that *HbS* contributes to resistance to expansion of blood stage parasite irrespective of malaria clinical presentation. Indeed, these results sustain the debate around the effect of *HbS* protection against hyperparasitaemia and its mechanisms. An experimental cerebral malaria (ECM) study in mice expressing sickle haemoglobin concluded that the protective effect exerted against lethal ECM was irrespective of parasite load [25]. Meanwhile, a human study evidenced that median parasite densities were significantly higher in *APOE* ε4 children for *Plasmodium* spp. densities compared to non-*APOE* ε4 children and concluded a suggestive epistatic interaction between *APOE* and *HbS* genes such that sickle cell trait only had an effect on parasite density in *APOE ε4* children [27].

The predominant occurrence of severe malaria in children under 5 years of age in the study sample is in line with what is reported in other areas of stable malaria [29, 30]. It has been argued, that lower frequency of severe malaria as well as decreased parasitaemia at both extremes of the age distribution could reflect, on one hand, the protective role of maternal immunity in children under 18 months and, on the other hand, the acquisition of immunity with age in children older than 9 years [31, 32]. Nevertheless, it cannot be ruled out that uneven representation of hyperparasitaemia and severe malaria across the distribution age may have an impact on *HbS* protection effects here identified.

The *HbS* allele frequency in the uninfected control group was 15.1 %, similar to that of the HapMap Yoruba (Nigeria) sample where the frequency is 12.5 %, though *HbS* frequency may exceed 20 % in other African populations [33]. Case–control analysis demonstrated a stronger protective effect of the *HbS* allele against severe malaria syndromes (OR = 0.15) as compared to uncomplicated malaria (OR = 0.50). Studies in Kenya reported similar genetic effects on protection against uncomplicated malaria (OR = 0.50) and against severe malaria (OR = 0.17) [6]. This strong protection against severe malaria conferred by the heterozygous HbAS was also clearly confirmed with a case–control study of 2591 severe falciparum malaria children enrolled at a tertiary referral center in Ghana (OR = 0.08) [34]. Another study that followed a cohort of 1070 children in Ghana showed protection against high parasitaemia in uncomplicated malaria [35]. It is worth mentioning that similarly to this Angolan sample, the protective effect of the *HbS* allele was evaluated in case–control supplemented with TDT [36] and was not diluted in a genome-wide association study of severe malaria, despite the ethnic diversity of the Gambian population [2].

The results of the present study reinforce the notion that parasitaemia levels are to be taken into account on evaluation of *HbS* protection in severe malaria. Nevertheless, the mechanism of *HbS* protection against hyperparasitaemia was not addressed in this study leaving open the possibility that *HbS* may partially accelerate the process of immunity acquisition against *P. falciparum* [37].

## Conclusion

Although these findings should be evaluated with caution due to sample size, they suggest that *HbS* does not significantly reduce the risk of developing clinical forms of severe malaria syndrome not entailing hyperparasitaemia and that the risk of co-occurrence of cerebral malaria or severe anaemia and hyperparasitaemia is higher in non-carriers. Thus, this study supports the hypothesis that *HbS* confers resistance to hyperparasitaemia in patients exhibiting severe malaria syndromes.
